# Liver Stiffness, Not Fat Liver Content, Predicts the Length of QTc Interval in Patients with Chronic Liver Disease

**DOI:** 10.1155/2019/6731498

**Published:** 2019-12-23

**Authors:** Mattia Bellan, Cristina Rigamonti, Greta Maria Giacomini, Giulio Makmur, Cecilia Marconi, Francesco Nicosia, Antonio Panero, Carla De Benedittis, Michela E. Burlone, Rosalba Minisini, Mario Pirisi

**Affiliations:** ^1^Department of Translational Medicine, Università del Piemonte Orientale (UPO), via Solaroli 17, 28100 Novara, Italy; ^2^Division of Internal Medicine, “Maggiore della Carità” Hospital, Corso Mazzini 18, 28100 Novara, Italy

## Abstract

The severity of fatty liver at ultrasound has been associated with QT length, a finding invoked to explain the excess cardiovascular risk of patients with fatty liver. However, the ability of ultrasound to stage accurately the severity of fatty liver is limited, with fibrosis a major confounder. Here, we aimed to verify the alleged relationship between fat liver content and QT length using a technique apt at discriminating steatosis from fibrosis noninvasively, i.e., transient elastography (TE) with measure of liver stiffness (LS) and controlled attenuation parameter (CAP). A prospectively collected derivation cohort of 349 patients with chronic liver disease (CLD) of any etiology (*N* = 105 with nonalcoholic fatty liver) was studied to identify clinical, laboratory, and instrumental predictors of the corrected QT interval (QTc) and QTc prolongation, including LS and CAP. The results were validated on a subgroup of patients belonging to the derivation cohort (out of sample validation), as well as on a completely different group of *N* = 149 subjects with CLD (out of time validation). QTc values were directly related to liver stiffness (LS; *ρ* = 0.137; *p* = 0.011), heart rate (HR; *ρ* = 0.307; *p* < 0.001), and age (*ρ* = 0.265; *p* < 0.001) and were significantly longer in females (*p* < 0.001). In contrast, QTc was not associated with the value of controlled attenuation parameter (*ρ* = 0.019; *p* = 0.718); moreover, no discernible differences in QTc length were noted based on CLD etiology. QTc was prolonged in 24/349 patients (6.9%); age, HR, and LS were independent predictors of QTc prolongation (*χ*^2^ = 23.7, *p* < 0.001). Furthermore, QTc values (after logarithmic transformation) were predicted by a model including age, gender, HR, and LS (*F* = 14.1, *R*^2^ = 0.198, *p* < 0.001). These latter results were validated by both out-of-sample and out-of-time methods. In conclusion, TE findings strongly suggest that among patients with CLD, fibrosis, not steatosis, is a major determinant of QTc length.

## 1. Introduction

Nonalcoholic fatty liver disease (NAFLD) is an important health concern, being on a global scale the most prevalent chronic liver disease (CLD). Its severity ranges from simple steatosis, to nonalcoholic steatohepatitis (NASH) with or without fibrosis, to cirrhosis [1]. Most of NAFLD patients, however, do not die because of the liver disease itself but of heart disease [2]. Indeed, NAFLD has been associated to an increased risk of fatal and nonfatal cardiovascular diseases [3] and is an independent predictor of atrial fibrillation [4]. Interestingly, the presence and severity of findings consistent with a fatty liver on ultrasound are associated with a prolonged corrected QT (QTc) interval both among patients with type 2 diabetes mellitus [5] and in the general population. Since QT length predicts cardiovascular death even when it is within limits considered normal, it is tempting to attribute to its prolongation part of the excess cardiovascular mortality of NAFLD patients. The mechanisms underlying the association between QT length and fatty liver are unclear, though they seem to be independent of traditional cardiometabolic factors and of systemic inflammation [6].

The development of CLD is often complicated by alterations in other organs and systems, including the heart. The so-called cirrhotic cardiomyopathy, defined as a chronic cardiac dysfunction observed in patients with cirrhosis, is characterized by blunted contractile responsiveness to stress, and/or altered diastolic relaxation with electrophysiological abnormalities, in the absence of any other known cause of cardiac disease [7]. One of the major electrophysiological abnormalities of cirrhotic cardiomyopathy is QTc prolongation [8]. It is important to note that, in the presence of fibrosis, the specificity of ultrasound in identifying fatty liver is substantially diminished [9]; therefore, the alleged association between length of the QT interval and NAFLD would be better explored by simultaneously quantifying fibrosis and steatosis, which is not feasible with conventional ultrasound. Demonstrating that NAFLD patients have significantly longer QTc intervals when compared to patients with CLD of different etiologies would give further support to the true existence of this association.

Based on these premises, in the present paper, we aimed to investigate the relative strength of the association of fibrosis and steatosis with QTc prolongation, both in NAFLD and in chronic liver diseases of different etiologies with or without evidence of fatty liver. To fulfil these aims, in a cohort of CLD patients, we quantified noninvasive steatosis and fibrosis at the same time the QTc interval was measured. Using these data, we developed and validated a model to predict the length of the QTc interval in such patients.

## 2. Materials and Methods

Our research was conducted according to the principles of the Declaration of Helsinki. All the procedures were performed in clinical practice and, therefore, a specific approval from the Ethical Committee was not required. However, every patient signed an informed consent for the anonymous distribution of data for research proposal.

Firstly, a cross-sectional derivation cohort was identified among consecutive patients attending the liver clinic of a university hospital. We included adult patients (older than 18 years), who received a diagnosis of chronic liver diseases from any etiology. We excluded patients affected by atrial fibrillation, atrial flutter, atrioventricular block, and right or left bundle block; those who had experienced a myocardial infarction; those who had undergone pacemaker implantation; and those who were receiving drugs known to prolong the QTc interval or antiarrythmic drugs. Patients receiving “conditional risk” drugs were allowed; “conditional risk” drugs are potentially responsible of QTc prolongation but only under specific circumstances (i.e., drug overdose, drug interaction, or in case of long QT syndrome).

After the application of the inclusion and exclusion criteria, we identified 349 patients; 105 were affected by NAFLD, 200 by chronic viral hepatitis, 12 by alcoholic liver disease, 3 by inherited diseases, 14 by autoimmune hepatitis and, finally, 16 by cryptogenic liver diseases.

A validation cohort of 149 patients was further selected, according to similar inclusion and exclusion criteria; out of them, 50 were affected by NAFLD, while 99 were affected by chronic viral hepatitis.

The demographic and clinical data collected included age, gender, smoking and alcohol habits, past medical history, and concomitant treatment(s). Moreover, all patients underwent a thorough physical examination. The body mass index (BMI) was calculated as body weight in kg (measured with the patient wearing light underwear) divided by the square of the height in meters and interpreted according to the World Health Organization classification [10]. The waist circumfetence (WC) was measured midway between the lowest rib and the iliac crest when standing. Blood pressure was measured after a period of relaxation in the seated position using a manual sphygmomanometer.

All the patients underwent a transient elastography examination by FibroScan (Echosens, Paris, France), after at least 6 hours of fasting. This exam was performed by a single expert hepatologist, blinded to the results of ECG, as previously reported [11]. The rate of successful measurements was calculated as the ratio of validated measurements to total measurements. The examination was considered reliable if at least 10 validated measurements were obtained for the patient with a greater than 65% success rate and if the interquartile range of all validated measurements was lower than 30% of the median value. The median value of successful measurements was considered to be representative of liver stiffness (LS) and was expressed in kilopascals. Liver fibrosis was ruled out in case of LS < 6 kPa. Moreover, all patients included in the derivation cohort also underwent a measurement of the controlled attenuation parameter (CAP), which allows noninvasive semiquantitative assessment of liver fat content by measuring the attenuation at the center frequency of the FibroScan® probe, ensuring that the liver ultrasonic attenuation was obtained simultaneously from the same volume of liver parenchyma as the LS. CAP values range from 100 to 400 dB/m: the cutoff values we chose to indicate steatosis as absent, mild, moderate, and severe were <215 dB/m, ≥215 dB/m, ≥252 dB/m, and ≥296 dB/m, respectively [12].

A 12-lead ECG trace (length: 10 seconds) was recorded by a trained nurse with the participant in the supine position on the morning of the same day of the visit. The QTc value was obtained recording the electrocardiogram by an interpretive machine (600G, Contec, No.112 Qinhuang West Street, Economic &Technical Development Zone, Qinhuangdao, Hebei Province, 66000, China) and was considered normal when <450 msec in males and <470 msec in females [13]. Resting heart rates were obtained from ECG readings.

## 3. Statistical Analysis

Anthropometric, clinical, and biochemical data were recorded in a database and analyzed by the statistical software package Stata, version 15.1 (StataCorp LP, College Station, Texas, US). The measures of centrality and dispersion of data chosen were medians and 95% CI. Medians were compared between groups by the Mann–Whitney and Kruskal–Wallis (K–W) tests. Exact Fisher's test and Pearson's *χ*^2^ test were used, as appropriate, to explore the associations of categorical variables. Cuzick's test was used to test the trend of a continuous variable between groups [14]. The association between continuous variables was tested by Spearman's correlation. Models were built to predict a set of explanatory variables: (a) a significantly prolonged QTc (by logistic regression analysis) and (b) the actual QTc value (multiple linear regression analysis; due to nonnormal distribution, the dependent variable had to be transformed logarithmically). Then, we performed an *out-of-sample validation* of the regression model, by evaluating whether the results obtained were similar when the model was applied to two subsamples of *N* = 175 and *N* = 174 patients randomly selected from the derivation cohort (samples A and B, respectively). The concordance between predicted and measured QTc was verified in a Bland-Altman plot. Furthermore, we performed an *out-of-time validation*, by applying the model obtained from the derivation cohort to the validation cohort. The concordance between measured and expected QTc was also tested by Bland-Altman analysis. We calculated Lin's concordance coefficient between measured and expected QTc for both the validation methods.

The level of significance chosen for all statistical analysis was 0.05 (two-tailed).

## 4. Results

### 4.1. Derivation Cohort

The derivation cohort included 349 patients, whose main features are listed in Table 1.

In [Supplementary-material supplementary-material-1], we report the proportion of patients affected by cirrhosis and/or severe steatosis, according to the etiology of liver disease. Among NAFLD patients, there was a significantly higher prevalence of severe steatosis (47/105, 44.7% vs. 26/244, 10.6%; *p* = 0.0001) and a lower prevalence of cirrhosis (2/105, 1.9% vs. 27/244, 11.0%; *p* = 0.002). As shown in Table 2, QTc is significantly longer in females than in males, as expected, and lengthens above a LS threshold strongly suggestive of cirrhosis.

As shown in Table 3, the median QTc was not different between patients affected by NAFLD and those affected by CLD of different etiologies. On the contrary, NAFLD showed a significantly higher CAP and a significantly lower LS.

The QTc values were directly correlated to LS (*ρ* = 0.137; *p* = 0.011), heart rate (*ρ* = 0.307; *p* < 0.001), and age (*ρ* = 0.265; *p* < 0.001). The relationship between LS and QTc was strengthened excluding patients with NAFLD (*N* = 244; *ρ* = 0.225; *p* < 0.001), while it was not confirmed when only the NAFLD group was considered (*N* = 105, *ρ* = −0.038; *p* = 0.697). On the contrary, QTc values had no correlation with fat liver content as assessed by CAP, neither considering the entire derivation cohort (*ρ* = 0.019; *p* = 0.701) nor the NAFLD group (*ρ* = −0.038; *p* = 0.701) or all other patients (*ρ* = 0.051; *p* = 0.428). Furthermore, in NAFLD patients, where the prevalence of LS values above a threshold strongly suggestive of cirrhosis was only 1.9%, QTc was similar when categorizing patients according to the presence or absence of cirrhosis and the presence or absence of severe steatosis. By contrast, in patients with CLD of other etiologies, QTc was significantly longer among those with cirrhosis, independent of the presence/absence of severe steatosis (also see [Supplementary-material supplementary-material-1]).

QTc was prolonged in 24/349 patients (6.9%); out of them, 18 (75%) were affected by viral hepatitis, 5 (21%) by NAFLD, and 1 (7%) by alcoholic liver disease. In [Supplementary-material supplementary-material-1], we report the main features of the subjects showing a prolonged QTc. Among these, there was a significantly higher proportion of cirrhotic patients (25.0% vs. 7.1%, *p* = 0.009), but a similar proportion of patients with severe steatosis (33.3% vs. 21.0%; *p* = 0.12).

In a multivariable logistic regression analysis, age, heart rate, and LS were confirmed as independent predictors of QTc prolongation (*χ*^2^ = 23.7, *p* < 0.001; Table 4(a)); we also built a multiple linear regression model to predict the QTc value (after logarithmic transformation) (Table 4(b)). We managed to build a model predictive of QTc value including age, gender, heart rate, and LS (*F* = 14.1, *R*^2^ = 0.198, *p* < 0.001).

### 4.2. Out-of-Sample Validation

To confirm the results obtained from the derivation cohort, we randomly selected 175 patients from the same cohort (sample A) and we evaluated whether the results of the multiple linear regression might be replicated in this subgroup of subjects. As shown in Table 5, the results were similar (*F* = 10.2, *R*^2^ = 0.268, *p* < 0.001).

**Figure 1:**
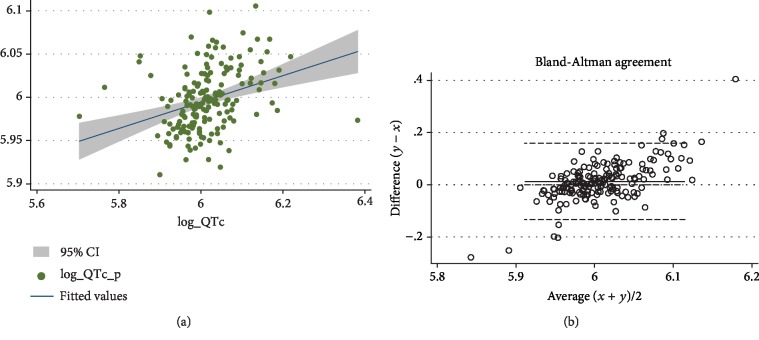
Concordance between the predicted and measured QTc values in sample B. In (a), we report the scatterplot of the predicted vs. measured QTc values (after logarithmic transformation); in (b), we report the Bland-Altman agreement plot. For both graphs, predicted QTc is on the *y*-axis, while measured QTc is on the *x*-axis.

We used the equation obtained from the analysis reported in Table 4 to calculate the log-transformed QTc values in the remaining 174 patients (sample B) of the derivation cohort. The predicted QTc was significantly associated to the measured QTc in the sample B patients (*ρ* = 0.417; *p* < 0.001; Figure 1(a)), as confirmed by Bland-Altman analysis (Figure 1(b)).

### 4.3. Out-of-Time Validation

To further validate our model, we applied the equation derived from the derivation cohort to a validation cohort. The validation cohort was composed by 149 patients (95 males, 63.8%), median age 60 (50-74) years. Fifty patients (33.6%) had NAFLD and 99 (66.4%) chronic viral hepatitis. The median BMI was 26.2 (23.6-29.9) kg/m^2^.

**Figure 2:**
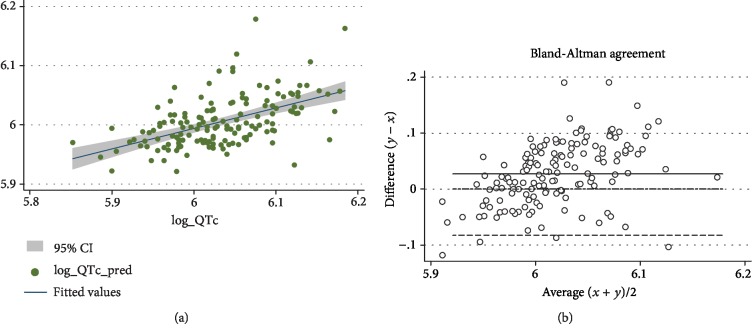
Concordance between the predicted and measured QTc values in the validation cohort. In (a), we report the scatterplot of the predicted vs. measured QTc values (after logarithmic transformation); in (b), we report the Bland-Altman agreement plot. For both graphs, predicted QTc is on the *y*-axis, while measured QTc is on the *x*-axis.

As shown in Figure 2(a), the measured QTc and the predicted QTc were significantly associated (*ρ* = 0.533; *p* < 0.001), as further confirmed by Bland-Altman plot (Figure 2(b)).

## 5. Discussion

QTc prolongation has been reported both in patients with cirrhosis and in those with nonalcoholic fatty liver disease. In the present study, we provide data suggesting that liver fibrosis is a stronger determinant of QTc prolongation than fatty liver, independent of the etiology of chronic liver disease. These findings need to be discussed at the light of the existing literature on this topic and of the limitations of the noninvasive techniques employed to determine the presence/absence of fatty liver and fibrosis.

Among similar classes of liver stiffness, we found similar QTc values independent of the severity of liver steatosis and the etiology of liver disease suggesting that, among patients with liver disease, QTc prolongation is not confined to a specific etiology. This is in line with previous reports from other groups [8, 15]. The fact that QTc is not different between conditions known to have a different prevalence of steatosis indirectly suggests that fatty liver might not be relevant in QTc prolongation [16]. At variance with the findings observed by others with an ultrasound method [5], CAP-assessed fat liver content bore no relationship to the length of the QTc interval. There could be several explanations for this discrepancy, including the different ethnic backgrounds of the populations studied and/or the subtlety of the association, which may require a very large sample size to be detected. It is important to note, however, that the estimate of liver fat content obtained by measuring the CAP is more objective than that obtainable by ultrasound. Besides, the mechanism underlying this postulated association is unknown yet. It has been proposed that QTc prolongation could be the result of an overactivation of sympathetic system [17]; since this issue was not specifically investigated in our paper, we cannot confirm or refute this hypothesis. However, it has long been known that sympathetic activation is common in compensated cirrhosis [18]. Here, we show that a proxy measure of liver fibrosis, LS measured by transient elastography is independently related to QTc length. Even if LS was previously shown to correlate not only with liver fibrosis but also with other histological figures like necroinflammatory activity, it reflects liver damage: the more severe liver damage is, the higher LS is. It must be said that the association between LS and QTc is rather weak, when considering the entire study population; however, it is influenced by CLD etiology, becoming stronger when NAFLD patients are excluded from the analysis (data not shown). Indeed, the association between LS and QTc was not observed in NAFLD patients, a fact that may be due to their (likely) lower necroinflammatory activity, their lower prevalence of cirrhosis, and/or the known difficulty in diagnosing cirrhosis by TE in this disease [19]. In the present study, QTc was found significantly longer in patients with liver stiffness equal or higher than 13 kPa, which many would consider a reliable cutoff for diagnosing cirrhosis [20]. This finding is in line with the observation that cirrhosis is associated with QTc prolongation. The association between LS and QTc is not limited to QTc prolongation in cirrhosis, though being extended to QTc values within normal reference values and minor degrees of LS elevation. In fact, LS is an independent predictor of QTc value, together with female gender, age, and heart rate, all well-known factors associated to the length of QTc [21–23]. In support of this hypothesis, we performed an out-of-sample and an out-of-time validation, both confirming the prediction model. On this basis, we speculate that liver steatosis is not related to QTc per se but rather through the development of liver fibrosis, an expected outcome of longstanding NAFLD with high liver fat content [24]. This is in line with studies that demonstrated a complex dysregulation of cardiovascular system in liver cirrhosis, leading to the definition of a relatively novel clinical entity, the so-called cirrhotic cardiomyopathy [25]. QTc prolongation is a relevant part of this syndrome, being highly prevalent in patients affected by liver cirrhosis [26] and, again, potentially explained by the increased adrenergic activity [27]. In fact, *β*-blockers reduce QTc length, while stressful events prolong it [28].

In the present study, we also managed to demonstrate that LS, along with HR and age, is a predictor of QTc prolongation. This is clinically relevant because it might help in identifying a subset of patients affected by chronic liver diseases who are at higher risk for significant QTc prolongation; specifically, a prolonged QTc is more likely in elderly women with advanced chronic viral hepatitis, a profile to be kept in mind before prescribing drugs that may prolong the QT interval. It is true, however, that sudden cardiac death is a relatively rare event in the natural history of cirrhosis; therefore, the clinical relevance of QTc prolongation in CLD remains to be established.

## 6. Conclusion

In conclusion, our data support the hypothesis of an association between QTc length and severity of liver damage in chronic liver disease and warn that the degree of fibrosis progression might be a major confounder to be taken into account when analyzing the relationship between QTc length and fatty liver.

## Figures and Tables

**Table 1:** Main demographic and clinical features of the derivation cohort. Continuous variables are shown as medians (IQR), while categorical variables are shown as frequencies (%).

Gender, *N*	
Males	193 (55.3%)
Females	156 (44.7%)
Age, years	63 (52 – 72)
Liver stiffness, kPa	6.0 (4.8 – 7.7)
CAP, dB/m	242 (213 – 285)
Etiology of liver disease	
NAFLD	105 (30.1%)
Chronic viral hepatitis	200 (57.3%)
Other etiologies	44 (12.6%)
BMI, kg/m^2^	25.7 (23.5 – 29.3)
<25	154 (44.1%)
25-29.9	124 (35.5%)
≥30	71 (20.4%)
Waist circumference, cm	98 (90 – 107)
Males	102 (94 – 109)
Females	94 (84 – 103)
T2DM, *N*	
No	279 (79.9%)
Yes	70 (20.1%)
Alcohol consumption, *N*	
No	202 (57.9%)
Regular consumption	119 (34.1%)
Regular alcohol abuse	27 (7.7%)
Binge drinking	1 (0.3%)
Cigarettes smoke, *N*	
Never smoked	146 (41.8%)
Previous smoker	138 (39.5%)
Current smoker, <15 cigarettes daily	39 (11.2%)
Current smoker, ≥15 cigarettes daily	26 (7.5%)
Physical activity, *N*	
Sedentary lifestyle	149 (42.7%)
<30 minutes of walk/day	96 (27.5%)
≥30 minutes of walk/day	104 (29.8%)

Abbreviations: BMI: body max index; CAP: controlled attenuation parameter; NAFLD: nonalcoholic fatty liver disease; QTc: corrected QT interval; T2DM: type 2 diabetes mellitus.

**Table 2:** QTc values in different categories of patients. The table shows the differences of QTc values in different categories of patients according to some relevant categorical and continuous variables. The median value of QTc is shown as median (IQR).

Variable	QTc (msec)	*p*
Gender		
Males (*N* = 193)	402 (387 – 420)	
Females (*N* = 156)	411 (397 – 438)	**<0.001**
BMI, kg/m^2^		
≤24.9 (*N* = 154)	409 (392 – 425)	
25.0 – 29.9 (*N* = 124)	407 (387 – 428)	
≥30.0 (*N* = 71)	406 (391 – 428)	0.609
T2DM		
No (*N* = 279)	407 (390 – 426)	
Yes (*N* = 70)	410 (396 – 430)	0.269
LS, kPa		
<5.9 (*N* = 163)	406 (388 – 426)	
5.9-12.9 (*N* = 157)	406 (391 – 425)	
≥13 (*N* = 29)	425 (409 – 461)	**0.013**
CAP (dB/m)		
<215 (*N* = 94)	408 (393 – 423)	
215-296 (*N* = 182)	406 (391 – 428)	
≥296 (*N* = 62)	408 (390 – 433)	0.434

Abbreviations: QTc: corrected QT; T2DM: type 2 diabetes mellitus; BMI: body mass index; LS: liver stiffness; CAP: controlled attenuation parameter.

**Table 3:** Differences according to CLD diagnosis. The table shows the differences according to the underlying cause of chronic liver disease. The continuous variables are shown as medians (IQR), while categorical variables are shown as *N* (%).

Variable	NAFLD	Other etiologies	*p*
LS, KPa	5.6 (4.5-6.7)	6.2 (5.0-8.0)	**0.0004**
CAP, dB/m	288 (259-334)	230 (203-263)	**<0.0001**
QTc, msec	408 (392-429)	407 (391-425)	0.60
CR drugs, N (%)	30 (28.6)	50 (20.5)	0.12

Abbreviations: LS: liver stiffness; CAP: controlled attenuation parameter; QTc: corrected QT; CR: conditional risk.

**(a):** 

Variable	Odds ratio (95% CI)	*p*
Age	1.044 (1.004 – 1.086)	**0.031**
Gender^∗^	0.828 (0.333 – 2.061)	0.685
HR	1.060 (1.017 – 1.104)	**0.005**
CR drugs^∗^	0.845 (0.299 –2.386)	0.751
LS	1.078 (1.022 – 1.136)	**0.006**
CAP	1.005 (0.997 – 1.012)	0.236

**(b):** 

Variable	Coefficient (95% CI)	*p*
Age	0.001 (0.000 – 0.002)	**<0.001**
Gender^∗^	-0.023 (-0.040 – -0.007)	**0.004**
HR	0.002 (0.001 – 0.003)	**<0.001**
CR drugs^∗^	0.010 (-0.008 – 0.003)	0.250
LS	0.003 (0.001 – 0.004)	**0.001**
CAP	0.000 (0.000 – 0.000)	0.433
Constant	5.786 (5.718 – 5.855)	**<0.001**

Abbreviations: HR: heart rate; CR: conditional risk; LS: liver stiffness; CAP: controlled attenuation parameter. The asterisk (∗) indicate variables that were entered into the model as categorical variables.

**Table 5:** Multiple regression analysis in the sample A. Herein, we show the results of the multiple linear regression of the QTc value (after logarithmic transformation) in a subgroup of 175 patients randomly selected from the derivation cohort.

Variable	Coefficient (95% CI)	*p*
Age	0.001 (0.000 – 0.002)	**0.002**
Gender	-0.037 (-0.060 – -0.015)	**<0.001**
HR	0.002 (0.001 – 0.003)	**<0.001**
CR drugs	0.009 (-0.002 – 0.004)	0.578
LS	0.003 (0.000 – 0.005)	**0.020**
CAP	0.000 (0.000 – 0.000)	0.761
Constant	5.786 (5.695 – 5.877)	**<0.001**

Abbreviations: HR: heart rate; CR: conditional risk; LS: liver stiffness; CAP: controlled attenuation parameter.

## Data Availability

The data used to support the findings of this study are available from the corresponding author upon request.

## Supplementary Materials

Supplementary Materials Table S1: distribution of cirrhosis and severe steatosis according to the etiology of liver disease. Liver cirrhosis was defined as liver stiffness = 13 kPa and severe steatosis as controlled attenuation parameter = 296 dB/m. Data are reported as number (row %). Abbreviations: CLD: chronic liver disease; NAFLD: nonalcoholic fatty liver disease. Table S2: differences of QTc values according to the etiology of liver disease, estimated fat liver content, and estimated degree of fibrosis. Liver cirrhosis is defined as liver stiffness = 13 kPa and severe steatosis for controlled attenuation parameter = 296 dB/m. ^∗^K-W test. Abbreviations: CLD: chronic liver disease; NAFLD: nonalcoholic fatty liver disease. Table S3: differences between patients with prolonged and normal QTc. The table shows the differences according to the underlying cause of chronic liver disease. The continuous variables are shown as medians (IQR), while categorical variables are shown as *N* (%). Abbreviations: BMI: body mass index; LS: liver stiffness; CAP: controlled attenuation parameter; QTc: corrected QT; CR: conditional risk.

## References

[1] Loria P., Adinolfi L. E., Bellentani S. (2010). Practice guidelines for the diagnosis and management of nonalcoholic fatty liver disease: A decalogue from the Italian Association for the Study of the Liver (AISF) Expert Committee. *Digestive and Liver Disease*.

[2] Targher G., Day C. P., Bonora E. (2010). Risk of cardiovascular disease in patients with nonalcoholic fatty liver disease. *The New England Journal of Medicine*.

[3] Targher G., Byrne C. D., Lonardo A., Zoppini G., Barbui C. (2016). Non-alcoholic fatty liver disease and risk of incident cardiovascular disease: a meta-analysis. *Journal of Hepatology*.

[4] Targher G., Valbusa F., Bonapace S. (2013). Non-alcoholic fatty liver disease is associated with an increased incidence of atrial fibrillation in patients with type 2 diabetes. *PLoS One*.

[5] Targher G., Valbusa F., Bonapace S. (2014). Association of nonalcoholic fatty liver disease with QTc interval in patients with type 2 diabetes. *Nutrition, Metabolism, and Cardiovascular Diseases*.

[6] Hung C.‐. S., Tseng P.‐. H., Tu C.‐. H. (2015). Nonalcoholic fatty liver disease is associated with QT prolongation in the general population. *Journal of the American Heart Association*.

[7] Møller S., Henriksen J. H. (2008). Cardiovascular complications of cirrhosis. *Gut*.

[8] Bernardi M., Calandra S., Colantoni A. (1998). Q-T interval prolongation in cirrhosis: prevalence, relationship with severity, and etiology of the disease and possible pathogenetic factors. *Hepatology*.

[9] Hernaez R., Lazo M., Bonekamp S. (2011). Diagnostic accuracy and reliability of ultrasonography for the detection of fatty liver: a meta-analysis. *Hepatology*.

[10] WHO Overweight and obesity fact sheet. http://www.who.int/mediacentre/factsheets/fs311/en/index.html.

[11] Sandrin L., Fourquet B., Hasquenoph J. M. (2003). Transient elastography: a new noninvasive method for assessment of hepatic fibrosis. *Ultrasound in Medicine & Biology*.

[12] de Lédinghen V., Vergniol J., Foucher J., Merrouche W., le Bail B. (2012). Non-invasive diagnosis of liver steatosis using controlled attenuation parameter (CAP) and transient elastography. *Liver International*.

[13] Goldenberg I., Moss A. J., Zareba W. (2006). QT interval: how to measure it and what is ‘normal’. *Journal of Cardiovascular Electrophysiology*.

[14] Cuzick J. (1985). A Wilcoxon-type test for trend. *Statistics in Medicine*.

[15] Mohamed R., Forsey P. R., Davies M. K., Neuberger J. M. (1996). Effect of liver transplantation on QT interval prolongation and autonomic dysfunction in end-stage liver disease. *Hepatology*.

[16] Lonardo A., Adinolfi L. E., Loria P., Carulli N., Ruggiero G., Day C. P. (2004). Steatosis and hepatitis C virus: mechanisms and significance for hepatic and extrahepatic disease. *Gastroenterology*.

[17] Liu Y. C., Hung C. S., Wu Y. W. (2013). Influence of non-alcoholic fatty liver disease on autonomic changes evaluated by the time domain, frequency domain, and symbolic dynamics of heart rate variability. *PLoS One*.

[18] Rector W. G., Robertson A. D. (1994). Prevalence and determinants of elevated plasma norepinephrine concentration in compensated cirrhosis. *The American Journal of Gastroenterology*.

[19] Singh S., Muir A. J., Dieterich D. T., Falck-Ytter Y. T. (2017). American Gastroenterological Association Institute technical review on the role of elastography in chronic liver diseases. *Gastroenterology*.

[20] European Association for Study of Liver, Asociacion Latinoamericana para el Estudio del Higado (2015). EASL-ALEH clinical practice guidelines: non-invasive tests for evaluation of liver disease severity and prognosis. *Journal of Hepatology*.

[21] Makkar R. R., Fromm B. S., Steinman R. T., Meissner M. D., Lehmann M. H. (1993). Female gender as a risk factor for torsades de pointes associated with cardiovascular drugs. *Journal of the American Medical Association*.

[22] Drew B. J., Ackerman M. J., Funk M. (2010). Prevention of torsade de pointes in hospital settings: a scientific statement from the American Heart Association and the American College of Cardiology Foundation. *Circulation*.

[23] Moss A. J., Schwartz P. J., Crampton R. S. (1991). The long QT syndrome. Prospective longitudinal study of 328 families. *Circulation*.

[24] Lallukka S., Sädevirta S., Kallio M. T. (2017). Predictors of liver fat and stiffness in non-alcoholic fatty liver disease (NAFLD) - an 11-year prospective study. *Scientific Reports*.

[25] Wiese S., Hove J. D., Bendtsen F., Møller S. (2014). Cirrhotic cardiomyopathy: pathogenesis and clinical relevance. *Nature Reviews Gastroenterology & Hepatology*.

[26] Teli M. R., James O. F., Burt A. D., Bennett M. K., Day C. P. (1995). The natural history of nonalcoholic fatty liver: a follow-up study. *Hepatology*.

[27] Zambruni A., Trevisani F., Caraceni P., Bernardi M. (2006). Cardiac electrophysiological abnormalities in patients with cirrhosis. *Journal of Hepatology*.

[28] Zambruni A., Trevisani F., Di Micoli A. (2008). Effect of chronic *β*-blockade on QT interval in patients with liver cirrhosis. *Journal of Hepatology*.

