# Supplementary material for: Liver Stiffness, Not Fat Liver Content, Predicts the Length of QTc Interval in Patients with Chronic Liver Disease
Source: Gastroenterol Res Pract. 2019 Dec 23;2019:6731498. doi: 10.1155/2019/6731498 (PMC6942798; doi:10.1155/2019/6731498)
Supplement: Supplementary Materials — Table S1: distribution of cirrhosis and severe steatosis according to the etiology of liver disease. Liver cirrhosis was defined as liver stiffness = 13 kPa and severe steatosis as controlled attenuation parameter = 296 dB/m. Data are reported as number (row %). Abbreviations: CLD: chronic liver disease; NAFLD: nonalcoholic fatty liver disease. Table S2: differences of QTc values according to the etiology of liver disease, estimated fat liver content, and estimated degree of fibrosis. Liver cirrhosis is defined as liver stiffness = 13 kPa and severe steatosis for controlled attenuation parameter = 296 dB/m. ∗K-W test. Abbreviations: CLD: chronic liver disease; NAFLD: nonalcoholic fatty liver disease. Table S3: differences between patients with prolonged and normal QTc. The table shows the differences according to the underlying cause of chronic liver disease. The continuous variables are shown as medians (IQR), while categorical variables are shown as N (%). Abbreviations: BMI: body mass index; LS: liver stiffness; CAP: controlled attenuation parameter; QTc: corrected QT; CR: conditional risk. [file 6731498.f1.pdf]

Supplementary material

CLD etiology	No cirrhosis No severe steatosis	No cirrhosis Severe steatosis	Cirrhosis No severe steatosis	Cirrhosis Severe steatosis	Total
**NAFLD**	58 (55.2)	45 (42.9)	0 (0.0)	2 (1.9)	105 (100.0)
**Others**	197 (80.7)	20 (8.2)	21 (8.6)	6 (2.5)	244 (100.0)
**Total**	255 (73.1)	65 (18.6)	21 (6.0)	8 (2.3)	349 (100.0)

Table S1. Distribution of cirrhosis and severe steatosis according to liver disease diagnosis. Liver cirrhosis has been defined for liver stiffness ≥ 13 kPa, severe steatosis for controlled attenuation parameter ≥ 296 dB/m. Data are reported as number (row %). Abbreviations: CLD= chronic liver disease; NAFLD= non alcoholic fatty liver disease.

CLD	No cirrhosis<br>No severe steatosis	No cirrhosis<br>Severe steatosis	Cirrhosis<br>No severe steatosis	Cirrhosis<br>Severe steatosis	p
**NAFLD**	408.5 (395-428)	406 (386-431.5)	-	412 (411-413)	0.89*
**Others**	403 (389-423)	407.5 (393-428)	425 (402-457)	447 (425-480)	**0.003***

Table S2. Differences of QTc values according to CLD etiology, liver steatosis and liver cirrhosis. Liver cirrhosis has been defined for liver stiffness ≥ 13 kPa, severe steatosis for controlled attenuation parameter ≥ 296 dB/m. *: K-W test. Abbreviations: CLD= chronic liver disease; NAFLD= non alcoholic fatty liver disease

Variable	Normal QTc<br>N.=325	Prolonged QTc<br>N.=24	p
**Age, years**	63 (52-72)	65 (54-74)	0.44
**Female gender, N (%)**	144 (44.3)	12 (50.0)	0.67
**BMI, kg/m^2**	25.6 (23.4-29.3)	27.5 (24.1-28.9)	0.31
**LS, kPa**	6.0 (4.8-7.6)	6.5 (5.0-13.1)	0.14
**LS >12.9 kPa, N (%)**	23 (7.1)	6 (25.0)	**0.009**
**CAP, dB/m**	241 (212-282)	252 (224-311)	0.28
**CAP ≥296 dB/m, N (%)**	65 (20.0)	8 (33.3)	0.12
**QTc, msec**	405 (390-423)	475 (464-489)	**<0.0001**
**CR drugs, N (%)**	50 (20.5)	30 (28.6)	0.12

Table S3. Differences between patients with prolonged and normal QTc. The table shows the differences according to the underlying cause of chronic liver disease. The continuous variables are shown as medians (IQR), while categorical variables are shown as N., (%). Abbreviations: BMI = Body Mass Index; LS = Liver Stiffness; CAP = Controlled Attenuation Parameter; QTc = corrected QT; CR, Conditional Risk.
